# A case of Huntington disease‐like 2 in a patient of African ancestry: the everlasting support of clinical examination in the molecular era

**DOI:** 10.1002/ccr3.6308

**Published:** 2022-10-06

**Authors:** Federica Ruscitti, Paola Origone, Giulia Rosti, Lucia Trevisan, Roberta Marchese, Andrea Brugnolo, Federico Massa, Paola Castellini, Paola Mandich

**Affiliations:** ^1^ Department of Neuroscience, Rehabilitation, Ophthalmology, Genetics, Maternal and Child Health (DINOGMI) University of Genoa Genoa Italy; ^2^ IRCCS Ospedale Policlinico San Martino, Medical Genetics Unit Genoa Italy; ^3^ IRCCS Ospedale Policlinico San Martino, Neurological Unit Genoa Italy; ^4^ IRCCS Ospedale Policlinico San Martino, Clinical Psychology Unit Genoa Italy; ^5^ IRCCS Ospedale Policlinico San Martino, Phoniatric Unit Genoa Italy

**Keywords:** brazilian ancestry, chorea, huntington disease‐like 2, *JPH3*

## Abstract

Chorea, cognitive decline, and psychiatric symptoms are shared by Huntington’s disease (HD) and similar conditions called HD phenocopies. We describe the first case reported in Italy of Huntington disease‐like 2 (HDL2), clinically and radiologically indistinguishable from HD, showing the importance of considering African ancestry in the diagnostic process.

## Graphical abstract

Given the strong clinical and radiological similarities between Huntington's disease (HD) and its phenocopies, we highlight the importance of a multidisciplinary clinical approach and the role of ethnicity in the diagnostic workout of chorea.

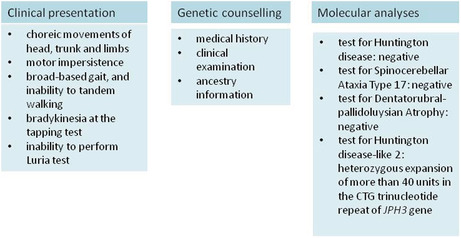

## 1 INTRODUCTION

Huntington’s disease (HD) is an autosomal dominant progressive neurodegenerative disease, characterized by movement disorders (mainly chorea, eventually combined with dystonia and bradykinesia), cognitive decline, and psychiatric symptoms. HD is caused by an expansion of more than 35 units in CAG trinucleotide repeat in the first exon of huntingtin gene (*HTT,* OMIM 613004).^1^ Patients showing all the cardinal clinical features of HD without the causative mutation in *HTT* are classified as HD phenocopies. So far, the genes described as associated with HD phenotypes include *TBP* (OMIM 600075), *JPH3* (OMIM 605268), *ATN1* (OMIM 607462), *C9ORF72* (OMIM 614260), and *PRNP* (OMIM 176640).^2^

We hereby describe the first case reported in Italy of Huntington disease‐like 2 (HDL2, OMIM 606438), caused by a CTG/CAG repeat expansion in the junctophilin 3 gene (*JPH3*).^3^

## 2 CASE HISTORY/EXAMINATION

The proband is a 51‐year‐old Brazilian woman who came to our attention due to the worsening of involuntary movements that began a few years before. Her familial history was negative for neurodegenerative diseases. At fifty years of age, the patient underwent surgical evacuation of a post‐traumatic subdural hematoma resolved without complications. On this occasion, during a phoniatric evaluation, the speech therapist first noticed involuntary movements. One year after surgery, the CT scan of brain was normal. A few months later, the patient underwent a neurological examination that showed broad‐based gait, inability to tandem walking, bradykinesia at the tapping test, inability to perform Luria test, motor impersistence, grimacing, and choreic movements of head, trunk, and limbs.These features led us to suspect a choreic disorder.

## 3 DIFFERENTIAL DIAGNOSIS AND INVESTIGATIONS

Clinical examination and blood tests ruled out nonhereditary causes of chorea, such as tardive dyskinesia, thyrotoxicosis, or cerebral lupus.

We first suspected HD, an autosomal dominant progressive neurodegenerative disease, characterized by movement disorders (mainly chorea, eventually combined with dystonia and bradykinesia), cognitive decline, and psychiatric symptoms. HD is caused by an expansion of more than 35 units in CAG trinucleotide repeat in the first exon of huntingtin gene (*HTT,* OMIM 613004).^1^ Since genetic testing for HD was negative, we considered other genetic causes of chorea.

Patients showing all the cardinal clinical features of HD without the causative mutation in *HTT* are classified as HD phenocopies. So far, the genes described as associated with HD phenotypes include *TBP*, *JPH3*, *ATN1*, *C9ORF72,* and *PRNP* ^2^.

On the clinical suspicion of an HD phenocopy, we performed analysis of *TBP* and *ATN1*, respectively, associated with spinocerebellar ataxia type 17 and dentatorubral‐pallidoluysian atrophy. Both the tests were negative.

A blood smear was performed by an expert hematologist to rule out acanthocytosis, and it was normal.

**FIGURE 1:**
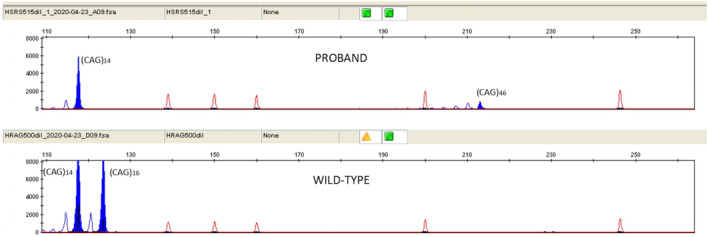
Molecular analysis of *JPH3* showing heterozygous expansion of more than 40 units in the CTG/CAG trinucleotide repeat, compared with control

Some patient's physical features (dark skin, frizzy hair, and broad flat nose) suggested an African ancestry. For this reason, we then took into account HDL2, which is usually indistinguishable from HD on neurological examination and typical of individuals with African ancestral background.^2^ HDL2 is an autosomal dominant condition caused by a CTG/CAG repeat expansion in the junctophilin 3 gene (*JPH3*). The normal allele size is between 6 and 28 repeats, and fully penetrant disease‐causing alleles show more than 40 repeats.^3^ The molecular analysis of *JPH3* detected in our patient an allele carrying 46 CTG/CAG trinucleotide repeat, confirming HDL2 diagnosis (Figure 1).

## 4 OUTCOME AND FOLLOW‐UP

**FIGURE 2:**
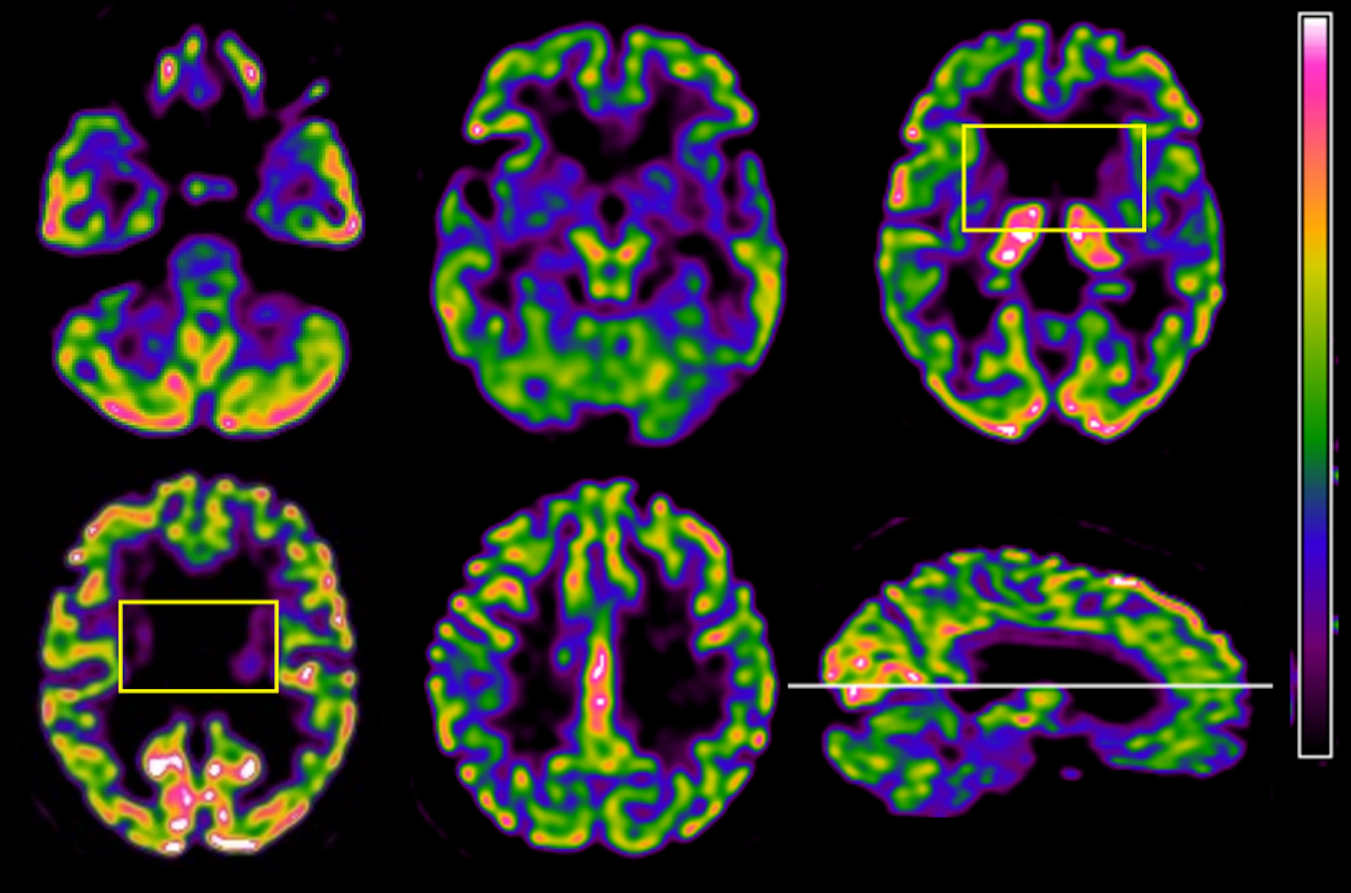
18F‐Fluorodeoxyglucose (FDG) PET images showing severe hypometabolism in bilateral basal ganglia (outlined boxes), but symmetrically preserved metabolism in midbrain, cerebellum, bilateral thalamic nuclei, and cortical areas

To assess the involvement of basal ganglia, we performed a fluorodeoxyglucose PET scan, which showed a reduction in glucose uptake in these structures (Figure 2).

On neuropsychological assessment, the patient was well oriented in time and space. Her global functioning level was lower than normal, with a mini‐mental state examination score of 21 out of 30 (normal values ≥24). Free and cued selective reminding test revealed that learning and memory abilities were relatively spared. The clock drawing test score of 1 (normal values ≥756) demonstrated loss of visuo‐spatial organization. Constructive apraxia test, performed for the evaluation of visuo‐constructional skills, and visual object and space perception battery (VOSP) were also impaired. The patient presented with a significant alteration of the executive function tasks and slow visuo‐spatial and language skills (assessed through symbol digit and stroop test).

A therapy with tetrabenazine 25 mg twice daily was set from the first neurologic evaluation to control involuntary movements.

One year after the diagnosis, the patient showed a rapid worsening of the psychiatric symptoms with aggressive behavior, psychomotor agitation, temporal disorientation, and refusal of any medical treatment. Due to an acute episode of agitation and confusion, she was admitted to the neurology department. On psychiatric assessment, she presented with speech disorganization, misperceptions, aggressive behavior, and temporal and spatial disorientation. The CT scan of brain did not show any hemorrhage and showed normal ventricular system and subarachnoid spaces.

The patient was transferred to the Psychiatric Diagnosis and Treatment Service. A therapy with intramuscular haloperidol 2 mg daily and intramuscular delorazepam 5 mg three times daily was initially set to control the psychomotor agitation. After discharge, a treatment with haloperidol 2 mg/mL 10 drops twice daily and delorazepam 1 mg/mL 10 drops three times daily was set as a maintenance therapy.

Speech and swallowing were gradually compromised over the course of the disease. The video‐recorded fibroendoscopic examination revealed slight incontinence of the velopharyngeal sphincter, normal morphology of the larynx, and the presence of sensitivity between the vocal cords. Swallowing of liquid, creamy, and solid food was tested through fiberoptic endoscopic evaluation (FEES), which showed a grade 5 dysphagia on the dysphagia outcome and severity scale (DOSS).

## 5 DISCUSSION

Huntington disease‐like 2 falls into the differential diagnosis of chorea, dementia, and psychiatric disorders. The clinical picture and the evidence of reduced glucose uptake in the basal ganglia (Figure 2) overlap with HD phenotype, although HDL2 generally presents an older age of onset, a higher frequency of parkinsonian features, and a lower rate of positive family history.^4^, ^5^ Instead, the occurrence of motor dysfunction as the first symptom and the suicidal ideation are more frequent in HD^6^. On macroscopic and microscopic neuropathological examination, HDL2 closely resembles HD. Even the pathogenesis of both diseases, although not fully understood, is likely to be mediated by both gain‐of‐function and loss‐of‐function mechanisms involving proteins and transcripts bidirectionally expressed from the expanded transcript.^7^

HDL2 is rare, with less than one hundred cases described worldwide, and it is nearly exclusive of individuals of African ancestry, including cases from Brazil and Venezuela.^8^, ^9^, ^10^ This is due to the fact that Latin America has a hybrid population, and in Brazil, the weighted mean proportions of European, African, and Native American genetic ancestries are estimated as 68.1%, 19.6%, and 11.6%, respectively.^11^ To the best of our knowledge, this patient is the first one reported in Italy, but some HDL2 cases have been described in the European population. In France, Mariani and colleagues found abnormal CTG/CAG expansion in *JPH3* in three patients among a cohort of 28 HD phenocopies. Out of these patients, two were of African descent, the third one was French Caucasian by mother and French West Indian by father.^2^

In conclusion, given the present multiethnicity of patients, clinicians might face conditions that are typical of different areas of the world. Therefore, it is fundamental to consider the ethnic backgrounds in the diagnostic workout. As far as HD and HD phenocopies are concerned, we suggest to take into account the research for *JPH3* expansion in a patient with chorea and likely African ancestry whose HD test is negative. Moreover, further investigations are indispensable to understand the shared pathogenic mechanisms underlying HD phenocopies, whose clinical phenotype and imaging findings show a high grade of similarity, suggesting a possible common target for future therapeutic strategies.

## AUTHOR CONTRIBUTIONS

Federica Ruscitti and Giulia Rosti involved in clinical examination and genetic counseling, and wrote the manuscript. Paola Origone involved in molecular testing. Lucia Trevisan revised the literature and manuscript. Roberta Marchese and Andrea Brugnolo involved in neurological examination. Federico Massa involved in imaging data management. Paola Castellini involved in phoniatric examination. Paola Mandich involved in clinical examination, genetic counseling, and clinical and molecular data management, and revised the manuscript.

## FUNDING INFORMATION

This research did not receive any specific grant from funding agencies in the public, commercial, or not‐for‐profit sectors.

## CONFLICT OF INTEREST

The authors have no actual or potential conflict of interest.

## ETHICAL APPROVAL

The authors state that no approval by ethics committee is required for the issue of this report.

## CONSENT

Written informed consent was obtained from the patient to publish this report in accordance with the journal's patient consent policy.

## PERMISSION TO REPRODUCE MATERIAL FROM OTHER SOURCES

Not applicable.

## CLINICAL TRIAL REGISTRATION

Not applicable.

## Data Availability

The datasets are available from the corresponding author on reasonable request.
